# An Account of Diseases in an Eastern District of London, from January 20, to February 20, 1808

**Published:** 1808-03

**Authors:** 


### *An Account of Diseases in an Eastern District of London, from January 20, to February 20, 1808.*

| | | | |
|---|---|---|---|
| ACUTE DISEASES. | | Phthisis Pulmonalis | 2 |
| Pneumonia | 2 | Hydrothorax | 1 |
| Peripneumonia Notha | 9 | Anasarca | 3 |
| Pleuritis | 2 | Gastrodynia | 3 |
| Variola | 1 | Dyspepsia | 4 |
| Rheumatismas Acutus | 3 | Hemiplegia | 3 |
| CHRONIC DISEASES. | | Dysuria | 5 |
| Tussis | 10 | Calculus | 1 |
| Dyspnœa | 8 | Amenorrhœa | 4 |
| Tussis cum Dyspnœa | 20 | Rheumatismus Chronicus | 12 |
| Hæmoptysis | 2 | PUERPERAL | |

| PUERPERAL DISEASES. | | INFANTILE DISEASES. | |
|---|---|---|---|
| Ephemera - - - - - - | 5 | Ophthalmia - - - - | 3 |
| Menorrhagia Lochialis - | 4 | Aphthæ - - - - - - | 2 |
| Hæmorrhois - - - - - | 3 | Herpes - - - - - - | 3 |

The case of small-pox mentioned in the list, presented a specimen of this disease in its most loathsome and alarming form. It was of the confluent kind, and the pustules almost completely covered the whole surface of the body. The restlessness of the child was so great as to render the use of opium necessary, in larger doses than usual.

The disease, however, yielded to the use of this and other remedies that were employed ; and in spite of every alarming symptom, terminated favourably.

On this occasion an additional proof was afforded of the effect produced by the late reports of the small-pox succeeding the use of vaccination. A considerable apprehension was excited amongst the neighbouring families, who, though their children had been vaccinated, and they had been informed by their medical friends, that the operation had succeeded to their wish, were yet with difficulty prevented from sending them to a distance, to avoid their being affected by the variolous contagion.

During the few last weeks the state of the weather has been such as that few constitutions have escaped its unpleasant effects. The cold on some days was so intense, and the transition from this to a milder state of the atmosphere so sudden, that it was natural to expect that some unfavourable consequences would ensue. Those affections of the lungs, which generally prevail at this season of the year have been considerably aggravated. An unusual number of colds, coughs, peripneumonies, and pleurisies have occurred, and these have been attended with dangerous and too frequently with fatal symptoms.

